# Association between serum vitamin A and body mass index in adolescents from NHANES 1999 to 2006

**DOI:** 10.1038/s41598-024-61437-0

**Published:** 2024-05-13

**Authors:** Xiaoqi Su, Nishant Patel, Shanliang Zhu, Xin Zhou, Ye Chen, Jun Chen, Xuming Mo

**Affiliations:** 1 Department of Ultrasound, Children’s Hospital of Nanjing Medical University, Nanjing, 210008 China; 2 Department of Cardiothoracic Surgery, Children’s Hospital of Nanjing Medical University, Nanjing, 210008 China; 3 School of Public Health, Nanjing Medical University, Nanjing, 211666 China

**Keywords:** Serum vitamin A, Obesity, Body mass index, NHANES, Adolescents, Population screening, Obesity

## Abstract

Vitamin A plays a pivotal role in health, particularly in regulating fat metabolism. Despite its significance, research into the direct relationship between vitamin A levels and obesity, especially among adolescents, is sparse. This study aims to explore this association within the adolescent population in the United States. This cross-sectional study analyzed the National Health and Nutrition Examination Survey (NHANES) data from 1999 to 2006, with 8218 participants. The levels of vitamin A in the serum were determined based on utilizing high-performance liquid chromatography with photodiode array detection. The relationship between serum vitamin A concentrations and body mass index (BMI) was evaluated using weighted multiple linear regression models, incorporating subgroup analyses by sex and race/ethnicity to provide nuanced insights. A positive correlation was observed between serum vitamin A levels and BMI, with BMI increasing progressively across vitamin A quartiles (P < 0.001). Using the lowest quartile of serum vitamin A as a reference, the BMI of the highest quartile of serum vitamin A was 1.236 times higher (95% CI 0.888, 1.585). Subgroup analyses revealed that this positive association persisted across different genders and racial/ethnic groups (P < 0.001). Notably, smooth curve fitting and saturation threshold analysis unveiled an inverted U-shaped relationship between serum vitamin A and BMI among female adolescents, non-Hispanic Whites, Mexican Americans, and other races/ethnicities groups. Our study substantiates the association between serum vitamin A levels and the risk of obesity/overweight status in adolescents. The findings suggest the potential serum vitamin A is an early biomarker for identifying obesity risk, although further studies are needed to determine to clarify its role as a contributing factor to obesity. This study contributes to the understanding of nutritional influences on adolescent obesity, highlighting the need for targeted interventions based on serum biomarkers.

## Introduction

Obesity among adolescents is increasingly recognized as a significant global health challenge, contributing to the heightened risk of developing chronic conditions such as diabetes and cardiovascular disease^1–3^. In clinical settings, anthropometric measures, particularly body mass index (BMI), are routinely employed to assess the risk of obesity. This metric has proven to be a reliable indicator of obesity in the adolescent population^4^.

The etiology of obesity during adolescence is multifaceted, including dietary, psychosocial, genetic, and environmental factors^5^. However, dietary habits are deemed to play a crucial role. Previous studies have highlighted the impact of poor dietary quality during adolescence, characterized by inadequate consumption of nutritious foods, such as fruits and vegetables^6,7^, and predilection of high-calorie, nutrient-poor options, such as hamburgers, cheeseburgers, and pizza^8,9^. Although the influence of macronutrients on adolescent obesity is well-documented, emerging evidence suggests that micronutrients, particularly vitamin A, also play a significant role^10,11^.

Vitamin A, a crucial fat-soluble vitamin, is indispensable for various aspects of human health and development. It is a vital dietary micronutrient involved in many physiological processes, including immune function, growth, and metabolism^12,13^. Studies have shown that vitamin A is critical in regulating fat metabolism, with implications for body weight management^14,15^.

Notably, a study by Libien et al. has underscored the involvement of vitamin A and its metabolites in the metabolism of adipose tissue^16^. Hypovitaminosis has been linked to an increased risk of obesity, influencing body weight regulation through its metabolic pathways^17,18^. Moreover, there appears to be an association between serum vitamin A levels and BMI, with obese individuals often exhibiting lower serum vitamin A concentrations compared to their normal-weight counterparts^19^. However, the studies^20^ in vitro and in vivo have further elucidated the role of vitamin A metabolites, such as retinal and retinoic acid, and their regulatory proteins in adipose tissue metabolism, contributing to the pathogenesis of obesity.

Although several studies have investigated the relationship between serum vitamin A levels and obesity, particularly in adolescent populations, remains inconclusive. This study aims to bridge the gaps in existing research by exploring the association between serum vitamin A levels and obesity among a large, diverse cohort of adolescents in the United States, spanning the years 1999 to 2006.

## Materials and methods

### Study population

This study leveraged data from the National Health and Nutrition Examination Survey (NHANES), a comprehensive national database that includes cross-sectional surveys covering demographic information, medical histories, prescription drug usage, and laboratory data, adhering to The Strengthening the Reporting of Observational Studies in Epidemiology (STROBE) guidelines.

**Figure 1:**
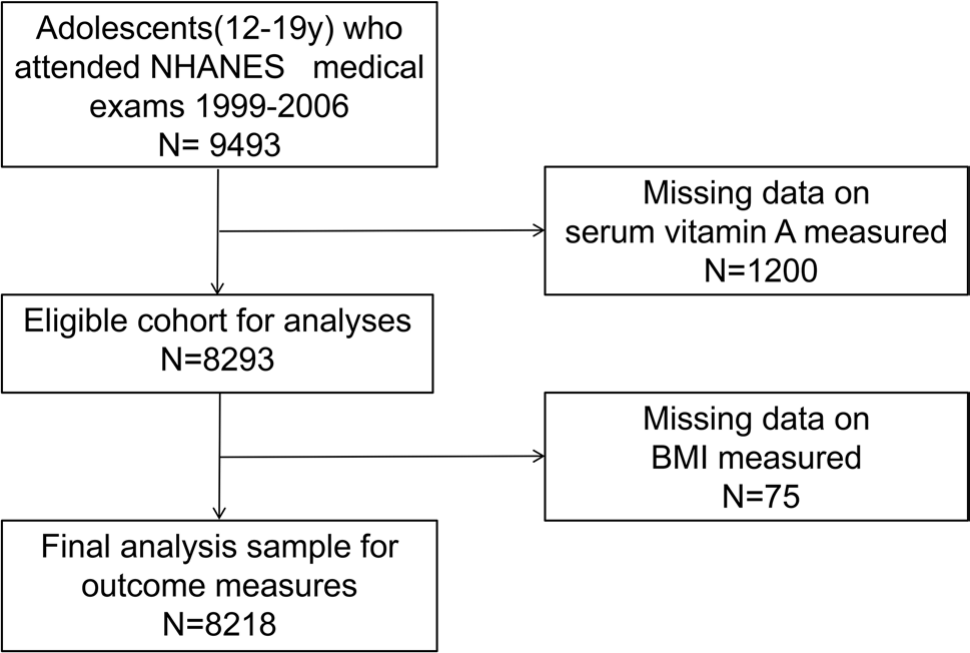
Eligible participants and those included in the analyses of the associations between serum vitamin A and body mass index in adolescents.

Data were extracted from four NHANES cycles spanning 1999 to 2006 (1999–2000, 2001–2002, 2003–2004, 2005–2006), selected for their inclusion of serum vitamin A data. The target population was adolescents aged 12–19 years (n = 9493), with complete records on serum vitamin A and BMI. Exclusions were made for missing data on serum vitamin A (n = 1200) and BMI (n = 75), resulting in a final sample size of 8218 participants for the retrospective cross-sectional survey analysis. The inclusion process is illustrated in Fig. 1.

The NHANES protocol was annually reviewed and approved by the National Center for Health Statistics (NCHS) Institutional Ethical Review Board, which also secured all participants' signed informed consent^21^. Participants aged 18 and above provided their own informed consent, while those under 18 participated with consent from a parent or guardian.

### Variables

Serum vitamin A levels and BMI served as the study's primary independent and dependent variables, respectively. Utilizing high-performance liquid chromatography with photodiode array detection, the levels of vitamin A in the serum were determined. Laboratory procedures and quality control methods for serum vitamin A measurement are described in detail elsewhere. The dependent variable, BMI was calculated from body measurements collected by trained health technicians, using the formula of weight in kilograms divided by height in meters squared (kg/m^2^).

Based on a review of the literature, potential covariates that could confound the relationship between serum vitamin A and BMI were summarized in multivariable-adjusted models. The demographics assessed in this study included age (years), sex (male/female), race/ethnicity (Mexican American, Other Hispanic, non-Hispanic White, Non-Hispanic Black, Other Race-including Multi-Racial), and poverty income ratio (PIR). Additional data collected at the medical examinations included laboratory, anthropometric, and questionnaire data. Blood samples were analyzed for total cholesterol, serum glucose, sodium, and potassium levels, and blood pressure was measured three times to obtain average systolic and diastolic values. Physical activity was measured using a specific “physical activity and physical fitness questionnaire” and included questions related to daily activities, leisure time activities, and sedentary activities at home. At the mobile inspection center, participants reported their physical activities and calculated metabolic equivalents in the past 30 days. Here, physical activity is divided into four categories: for instance, sedentary people are those who do not engage in regular physical activity. Low activity is defined as people with < 500 Metabolic Equivalent for Task (MET) min activity/week. Moderate activity is defined as people with 500–1,000 MET-min activity/week, and high activity is defined as people with > 1000 MET-min activity/week. Further details on the measurement processes and data acquisition for serum vitamin A, BMI, and other covariates are available online at http://www.cdc.gov/nchs/nhanes/.

### Statistical analysis

Baseline characteristics were summarized as means ± standard deviation (SD) (normal distribution) for continuous variables and as percentages for categorical variables, employing population-weighted parametric and nonparametric tests as appropriate. Serum vitamin A concentrations were ranked from smallest to largest and divided into four equal quartiles for analysis, both as continuous and categorical variables. Differences among groups were assessed using the weighted Chi-square tests for categorical variables and the weighted one-way ANOVA for continuous variables. Weighted multiple linear regression models were used to estimate linear relationships between serum vitamin A and BMI after adjusting for potential confounders. Furthermore, a smooth curve fitting and a generalized additive model were used to examine the linear or nonlinear relationship between serum vitamin A and BMI. When nonlinearity was detected, we calculated the inflection point using a recursive algorithm for subsequent two-piecewise linear regression analysis. All analyses were performed using EmpowerStats (http://www.empowerstats.com) and P < 0.05 value was considered statistically significant.

### Ethics approval and consent to participate

This study analyzed the data from the public database of the National Health and Nutrition Examination Survey. The ethical review committee of the National Health Statistics Center gave ethical approval. The methods involved in this study are carried out in accordance with relevant guidelines and regulations (Helsinki Declaration). All subjects provided written informed consent before participating in the study.

## Results

In this retrospective cross-sectional analysis of the NHANES from 1999 to 2006, a total of 8218 participants met the inclusion criteria. The demographic and baseline characteristics of the study population are detailed in Table 1. Among all participating adolescents, the mean age was 15.43 ± 2.27 years old, of which 51.34% were boys and 48.66% were girls. Racial and ethnic composition consisted of 61.57% non-Hispanic White, 14.5% non-Hispanic Black, 11.07% Mexican Americans, and 12.86% other racial and ethnic backgrounds, including Multi-Racial individuals. The overall mean ± SD serum vitamin A concentration was 47.80 ± 11.66 µg/dL, and the ranges of four serum vitamin A quartiles (Q1, Q2, Q3 and Q4) were 11.50–37.89, 37.90–44.38, 44.39–51.92 and 51.93–149.00 µg/dL, respectively. The overall mean ± SD of BMI was 23.38 ± 5.58, and participants with higher serum vitamin A quartiles tended to exhibit increased BMI values (Q1: 22.21 ± 5.57, Q2: 22.66 ± 5.32, Q3: 23.71 ± 5.72 and Q4: 24.38 ± 5.44; p < 0.0001), indicating a potential relationship between vitamin A levels and BMI. Significant differences were observed across the serum vitamin A quartiles concerning demographic and health-related variables including age, sex, race/ethnicity, PIR, physical activity, total cholesterol, diastolic blood pressure, systolic blood pressure, serum glucose, serum sodium, and serum potassium (all p < 0.05). Notably, participants within the highest serum vitamin A quartile were predominantly male and of White ethnicity.

**Table 1:** Description of 8218 participants included in the present study.

Serum vitamin A (µg/dL)	All	Q1 (11.50–37.89) N = 2055	Q2 (37.90–44.38) N = 2036	Q3 (44.39–51.92) N = 2072	Q4 (51.93–149.00) N = 2055	*p*-value
Age (years)	15.43 ± 2.27	14.74 ± 2.27	14.97 ± 2.24	15.41 ± 2.20	16.23 ± 2.11	< 0.0001
Sex (%)						< 0.0001
Male	51.34	37.16	43.09	55.54	62.67	
Female	48.66	62.84	56.91	44.46	37.33	
Race/ethnicity (%)						< 0.0001
Whites	61.57	44.96	58.10	63.50	72.68	
Blacks	14.50	27.90	15.90	12.11	7.31	
Mexican Americans	11.07	13.76	11.98	10.70	9.07	
Other race/ethnicity	12.86	13.38	14.02	13.68	10.94	
Poverty income ratio	2.49 ± 1.58	2.21 ± 1.49	2.52 ± 1.61	2.50 ± 1.59	2.64 ± 1.57	< 0.0001
Physical activity(%)						< 0.0001
Sedentary	4.88	5.12	5.04	4.80	4.67	
Low	9.49	8.00	8.10	8.05	12.68	
Moderate	7.44	6.03	5.88	8.48	8.61	
High	19.15	12.31	15.23	19.06	26.39	
Not recorded	59.05	68.54	65.75	59.60	47.65	
Systolic blood pressure (mmHg)	109.50 ± 10.01	107.42 ± 9.30	108.13 ± 9.80	109.70 ± 9.89	111.63 ± 10.26	< 0.0001
Diastolic blood pressure (mmHg)	61.23 ± 11.48	60.79 ± 11.23	60.56 ± 11.22	61.00 ± 11.94	62.20 ± 11.36	< 0.0001
Total cholesterol (mg/dL)	161.64 ± 31.43	153.72 ± 27.99	157.02 ± 28.37	160.56 ± 31.65	170.94 ± 33.14	< 0.0001
Serum glucose (mg/dL)	86.54 ± 14.61	85.54 ± 11.23	86.85 ± 19.08	87.04 ± 12.83	86.50 ± 13.86	0.0139
Serum sodium (mg/dL)	139.17 ± 1.95	139.03 ± 1.92	139.10 ± 1.96	139.20 ± 1.92	139.29 ± 1.99	0.0003
Serum potassium (mmol/L)	4.03 ± 0.29	3.99 ± 0.30	4.02 ± 0.29	4.05 ± 0.29	4.06 ± 0.29	< 0.0001
BMI (kg/m^2^)^a^	23.96 ± 5.95 22.46 (13.14, 62.08), 48.94	23.15 ± 6.27 21.49 (13.14, 62.08), 48.94	23.58 ± 5.71 22.21 (13.43, 57.23), 43.80	24.15 ± 5.93 22.61 (13.85, 55.20), 41.35	24.95 ± 5.74 23.54 (14.14, 60.85), 46.71	< 0.0001

Description of 8218 participants included in the present study.

Mean ± SD for continuous variables: *p*-value was calculated by weighted one-way ANOVA analysis.

% for categorical variables: *p*-value was calculated by weighted chi-square test.

^a^BMI variable also presented as median (min, max) interquartile range.

**Figure 2:**
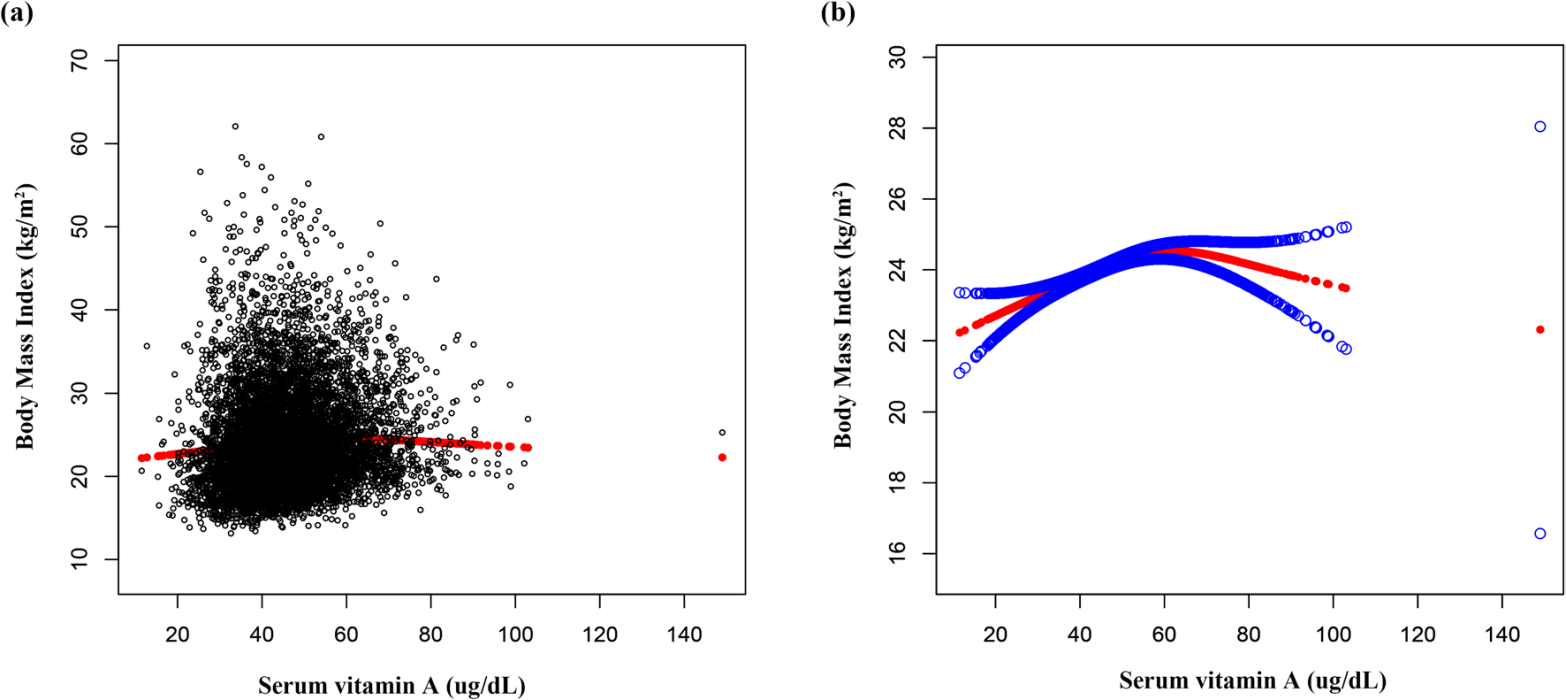
The association between serum vitamin A and body mass index. (**a**) In correlation plot, each black point represents a sample, illustrating the distribution of serum vitamin A levels across the study population. (**b**) In smooth curve plot, the area between two blue dotted lines is expressed as a 95% CI. Each point shows the magnitude of the serum vitamin A and is connected to form a continuous line. Age, sex, race/ethnicity, poverty income ratio, physical activity, systolic blood pressure, diastolic blood pressure, total cholesterol, serum glucose, serum sodium, and serum potassium were adjusted.

Multivariate linear regression analyses, as presented in Table 2, demonstrated a positive association between serum vitamin A levels and BMI in the unadjusted model (β = 0.067, 95%CI 0.057- 0.077). This positive correlation persisted in model 2 (β = 0.059, 95%CI 0.048–0.069), and model 3 (β = 0.032, 95%CI 0.021–0.042) (Fig. 2) even after adjusting for confounding factors in subsequent models. After transforming serum vitamin A into a categorical variable rather than a continuous one, individuals in the highest quartile had a 1.236 kg/m^2^ higher BMI than those in the lowest serum vitamin A quartile.

**Table 2:** Association of serum vitamin A with body mass index.

	Model 1 β (95% CI)	Model 2 β (95% CI)	Model 3 β (95% CI)
Serum vitamin A	0.067 (0.057, 0.077)	0.059 (0.048, 0.069)	0.032 (0.021, 0.042)
Serum vitamin A categories			
Q1	Reference	Reference	Reference
Q2	0.448 (0.079, 0.816)	0.631 (0.272, 0.991)	0.420 (0.084, 0.757)
Q3	1.498 (1.139, 1.857)	1.641 (1.284, 1.999)	1.202 (0.866, 1.539)
Q4	2.170 (1.821, 2.519)	2.072 (1.709, 2.434)	1.236 (0.888, 1.585)
Stratified by sex			
Male	0.107 (0.093, 0.122)	0.081 (0.066, 0.096)	0.044 (0.029, 0.059)
Female	0.036 (0.021, 0.052)	0.038 (0.023, 0.054)	0.023 (0.008, 0.038)
Stratified by race/ethnicity			
Whites	0.085 (0.066, 0.104)	0.053 (0.033, 0.073)	0.028 (0.009, 0.047)
Blacks	0.075 (0.049, 0.100)	0.066 (0.041, 0.091)	0.038 (0.013, 0.063)
Mexican Americans	0.077 (0.058, 0.096)	0.072 (0.052, 0.091)	0.032 (0.013, 0.052)
Other race/ethnicity	0.076 (0.038, 0.113)	0.073 (0.035, 0.111)	0.040 (0.004, 0.076)

Model 1, no covariates were adjusted.

Model 2, age, sex, race/ethnicity were adjusted.

Model 3, age, sex, race/ethnicity, income-poverty ratio, physical activity, systolic blood pressure, diastolic blood pressure, total cholesterol, serum glucose, serum sodium, and serum potassium were adjusted.

In the subgroup analysis stratified by sex or race/ethnicity, the model is not adjusted for the stratification variable itself.

Subgroup analyses (as shown in Table 2) further elucidated the relationship between serum vitamin A and BMI across different demographics. Positive correlations were evident in both male (β = 0.044, 95%CI 0.029–0.059) and female (β = 0.023, 95%CI 0.008–0.038), along with White (β = 0.028,95%CI 0.009–0.047), Black (β = 0.038, 95%CI 0.013–0.063), and Mexican Americans (β = 0.032, 95%CI 0.013–0.052), as well as other race/ethnicity (β = 0.040, 95%CI 0.004–0.076).

**Figure 3:**
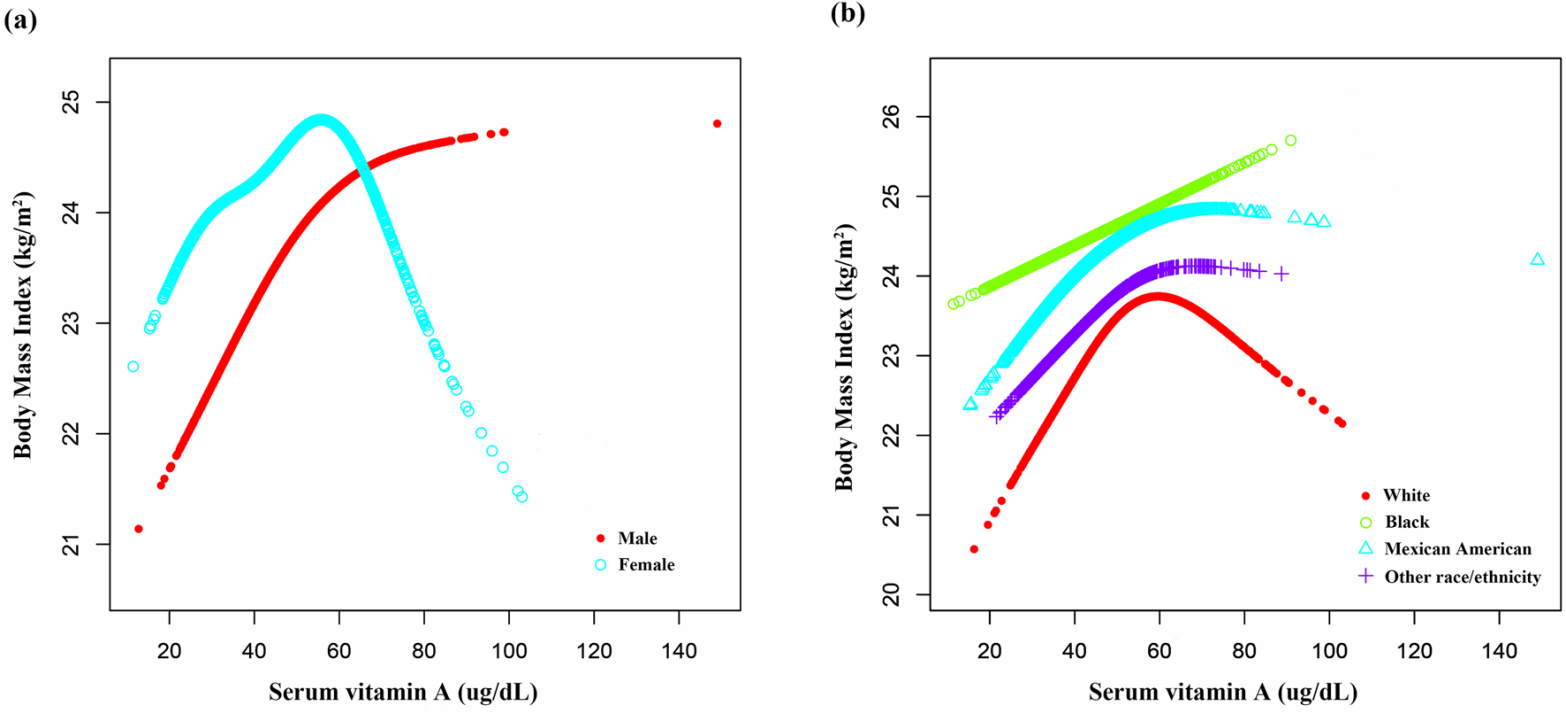
The association between serum vitamin A and body mass index. Smooth curve fitting plots showed the nonlinear relationships by stratification using sex (**a**) and race/ethnicity (**b**). Age, sex, race/ethnicity, poverty income ratio, physical activity, systolic blood pressure, diastolic blood pressure, total cholesterol, serum glucose, serum sodium, and serum potassium were adjusted.

In addition, to further explore the linear relationship between serum vitamin A and BMI in adolescents, smooth curve fitting and saturation threshold analysis (generalized additive models) were employed. The adjusted smoothed plots, stratified by sex and race/ethnicity, showed a nonlinear relationship between serum vitamin A and BMI (Fig. 3). As shown in Table 3, in adolescent females, BMI increased with serum vitamin A up to the turning point (serum vitamin A 53.7 µg/dL) (Fig. 3a). The turning point for Whites was serum vitamin A of 51.0 µg/dL (Fig. 3b), for Mexican Americans 41.2 µg/dL (Fig. 3b), and for other races/ethnicities 52.0 µg/dL (Fig. 3b). In brief, this nonlinear association between serum vitamin A and BMI was characterized by an inverted U-shaped curve in adolescent females, White, Mexican American, and other racial/ethnic groups.

**Table 3:** Association of serum vitamin A with body mass index.

Body mass index	Adjusted β (95% CI), p-value
Female	
Serum vitamin A < 53.7 (µg/dL)	0.085 (0.062, 0.108) < 0.0001
Serum vitamin A > 53.7 (µg/dL)	− 0.076 (− 0.107, − 0.044) < 0.0001
Whites	
Serum vitamin A < 51.0 (µg/dL)	0.095 (0.058, 0.133) < 0.0001
Serum vitamin A > 51.0 (µg/dL)	− 0.024 (− 0.055, 0.007) 0.1359
Mexican Americans	
Serum vitamin A < 41.2 (µg/dL)	0.099 (0.044, 0.153) 0.0004
Serum vitamin A > 41.2 (µg/dL)	0.011 (− 0.014, 0.037) 0.3838
Other race/ethnicity	
Serum vitamin A < 52.0 (µg/dL)	0.100 (0.043, 0.157) 0.0007
Serum vitamin A > 52.0 (µg/dL)	− 0.050 (− 0.126, 0.026) 0.1978

Threshold effect analysis of serum vitamin A on body mass index using two-piecewise linear regression.

Age, sex, race/ethnicity, income-poverty ratio, physical activity, systolic blood pressure, diastolic blood pressure, total cholesterol, serum glucose, serum sodium, and serum potassium were adjusted.

In the subgroup analysis for females, non-Hispanic whites, Mexican Americans, and other race/ethnicity, the model is not adjusted for sex or race/ethnicity, respectively.

## Discussion

This study aimed to investigate the relationship between serum vitamin A and BMI using a substantial and representative dataset from the NHANES database. The present study discerned a positive correlation between serum vitamin A concentrations and BMI, with a notable turning point in this linear relationship when participants were categorized into distinct groups.

Further stratification revealed that the impact of serum vitamin A on BMI varied across different demographics. Specifically, we observed a threshold effect in the relationship between serum vitamin A levels and BMI among female adolescents, contrasting with a substantial linear correlation in male adolescents. This distinction may be attributable to the differential susceptibility to hormonal influences between genders, as existing studies^22–24^ have shown a linkage between hormonal factors and obesity prevalence in female adolescents. To establish a definitive connection between gender-specific responses to serum vitamin A and BMI, further investigation through randomized controlled trials (RCT) is warranted.

Moreover, the study identified varying threshold effects in the association between vitamin A and obesity among White, Mexican American, and other race/ethnicity groups, with distinct response curves observed. This variability underscores the significant role of ethnicity in modulating obesity risk, potentially influenced by a myriad of socioeconomic factors, such as dietary habits, social behaviors, and familial socioeconomic status^25,26^. However, these findings contrast with previous research^27^ indicating higher obesity rates among African-American and Hispanic adolescents, suggesting that sample size disparities among ethnic groups in our study may have contributed to these differences.

Cardiovascular and metabolic complications are prevalent among obesity-related health issues^28–30^, highlighting the importance of understanding the role of nutritional biomarkers like serum vitamin A in obesity pathogenesis. Serum vitamin A is one of the serum biomarkers commonly used to assess the nutritional status of adolescents. Because serum vitamin A can be reliably detected, especially when the level of obese patients is significantly higher than that of normal people. Unfortunately, there is limited evidence of a link between serum vitamin A and obesity in adolescents. Our findings are consistent with a previous cross-sectional study of 3025 Eastern Chinese youths aged 7 to 17 by Ting Tian et al.^10^ documented that serum vitamin A was positively correlated with central obesity by logistic regression analysis. Besides, higher serum vitamin A in the oldest age group had observed higher BMI trends in our study. Similarly, previous studies have shown that age may be a highlighting factor that influences BMI due to various physiological and lifestyle factors, such as changes in body composition, metabolic changes, physical activity levels, nutritional changes, health conditions and medication use^31–33^. Furthermore, sex-stratified analyses showed that overall obesity risk still increased with increasing vitamin A levels in both male and female participants, which was contrary to our study. These contradictory conclusions may be attributed to the differences in demographic characteristics, research scale, and controllable confounding factors of these studies. Nonetheless, this conclusion needs to be further confirmed by large-sample, multicenter prospective studies.

The main advantage of this study lies in the analysis of a diverse, multi-ethnic sample, enabling detailed subgroup analyses. However, the retrospective cross-sectional design limits our ability to infer causality between vitamin A and BMI in adolescents aged 12–19. The potential effects of reverse causality, where dietary preferences such as high-fat^34^ and lesser vitamin-containing foods^35^ influenced by obesity status may affect serum vitamin A levels, cannot be discounted. Additionally, unaccounted confounding factors and the lack of data on visceral obesity or fatty liver disease among adolescents with normal BMI but increased visceral fat highlight the need for a cautious interpretation of the findings^36,37^. Meanwhile, this cross-sectional observational study data ranges from 1999 to 2006 years due to the unavailability of any recent data on vitamins in the NHANES database.

In conclusion, this study revealed a positive correlation between serum vitamin A levels and obesity in adolescents, with nuanced variation across genders and ethnic groups. The identification of serum vitamin A as a potential biomarker for early obesity detection and management invites further research to establish optimal serum vitamin A thresholds for obesity prevention and intervention strategies.

## Data Availability

The datasets generated and analyzed for the current study are available in the NHANES database. More information about the NHANES can be obtained at: https://wwwn.cdc.gov/nchs/nhanes/Default.aspx.

## Abbreviations

NHANES: National Health and Nutrition Examination Survey
BMI: Body Mass Index
